# Dendritic cell targeted Ccl3- and Xcl1-fusion DNA vaccines differ in induced immune responses and optimal delivery site

**DOI:** 10.1038/s41598-018-38080-7

**Published:** 2019-02-12

**Authors:** Anna Lysén, Ranveig Braathen, Arnar Gudjonsson, Demo Yemane Tesfaye, Bjarne Bogen, Even Fossum

**Affiliations:** 1 K.G. Jebsen Centre for Influenza Vaccine Research, Institute of Immunology, University of Oslo and Oslo University Hospital, Oslo, 0372 Norway; 2 Centre for Immune Regulation, Institute of Immunology, University of Oslo and Oslo University Hospital, Oslo, 0372 Norway

## Abstract

Fusing antigens to chemokines to target antigen presenting cells (APC) is a promising method for enhancing immunogenicity of DNA vaccines. However, it is unclear how different chemokines compare in terms of immune potentiating effects. Here we compare Ccl3- and Xcl1-fusion vaccines containing hemagglutinin (HA) from influenza A delivered by intramuscular (i.m.) or intradermal (i.d.) DNA vaccination. Xcl1 fusion vaccines target cDC1s, and enhance proliferation of CD4^+^ and CD8^+^ T cells *in vitro*. In contrast, Ccl3 target both cDC1 and cDC2, but only enhance CD4^+^ T cell proliferation in combination with cDC2. When Ccl3- or Xcl1-HA fusion vaccines were administered by i.m. DNA immunization, both vaccines induced Th1-polarized immune responses with antibodies of the IgG2a/IgG2b subclass and IFNγ-secreting T cells. After i.d. DNA vaccination, however, only Xcl1-HA maintained a Th1 polarized response and induced even higher numbers of IFNγ-secreting T cells. Consequently, Xcl1-HA induced superior protection against influenza infection compared to Ccl3-HA after i.d. immunization. Interestingly, i.m. immunization with Ccl3-HA induced the strongest overall *in vivo* cytotoxicity, despite not inducing OT-I proliferation *in vitro*. In summary, our results highlight important differences between Ccl3- and Xcl1- targeted DNA vaccines suggesting that chemokine fusion vaccines can be tailor-made for different diseases.

## Introduction

DNA vaccination represents a highly attractive immunization strategy due to the speed and cost effectiveness at which novel subunit vaccines can be generated. Unfortunately, the issue of low immunogenicity associated with DNA vaccination has been a significant challenge and, in many instances, hampered further clinical development. Different strategies have been developed in order to improve the immunogenicity of DNA vaccines, including electroporation (EP) of the injection site in order to enhance antigen expression^1^, or fusing antigens to chemokines or single-chain variable fragments (scFV) in order to target antigen presenting cells (APC)^2,3^. Further enhanced immunogenicity can be achieved by combining these two strategies, as we have previously demonstrated in a tumor model^4^ as well as an influenza virus infection model^5,6^.

Targeting antigen to APC is now a well-established method for enhancing immunogenicity. Since early attempts at targeting predominantly B cells by conjugation to anti-MHC-II^7^ or anti-immunoglobulin^8^, focus has generally shifted towards targeting more dendritic cell (DC) restricted receptors such as DEC205^9^ and Clec9A^10,11^. Several studies have demonstrated that targeting antigen to DCs enhances both T cell responses^10–12^ and antibody responses^10,13,14^.

A number of different chemokines and scFvs have been tested as fusion DNA vaccines, including CXCL10^2^, Ccl7^2^, Ccl3^4,5,15,16^, Ccl5^4,17^, Ccl20^18,19^, anti-DEC205^20^ and Xcl1^6,21^. However, there are few comparative studies where different fusion vaccines have been administered under similar conditions^2,4^. Since many chemokines bind several different chemokine receptors on multiple cell types, it is likely that the expression profiles of the targeted receptors influence the resulting immune response^22^. In addition, chemokines are biologically active molecules that induce different downstream signaling events, which may have adjuvant effects. Adding to the complexity of DNA vaccination and electroporation, the site of injection can also influence the resulting immune responses. The most common delivery routes of DNA +EP are intramuscular (i.m) or intradermal (i.d) injections. In terms of administration, i.d. immunization +EP is easier to administrate and is generally considered less painful compared to i.m. delivery +EP. However, studies have suggested that i.m. DNA vaccination, with or without EP, is more efficient at inducing cellular immune responses in both mice and macaques^23,24^.

In this study we compare the use of Xcl1 and Ccl3-fusion DNA vaccines for the induction of immune responses against influenza A hemagglutinin (HA). Xcl1 is a ligand of the receptor Xcr1, which is selectively expressed on conventional dendritic cell type 1 (cDC1)^25,26^. In contrast, Ccl3 acts as a ligand for the receptors Ccr1, Ccr3 and Ccr5 that are expressed on a number of different cell types including cDC1, cDC2, pDC, Langerhans cells, macrophages, NK cells and CD4^+^ and CD8^+^ T cells^27–32^. Consequently, Ccl3-fusion vaccines have a relatively broad tropism and target a number of different cell types that can act as APCs.

We here observe that DNA immunization with Ccl3- or Xcl1-fusion vaccines induce qualitatively different immune responses, and that the site of immunization influences the resulting immune responses. i.m. DNA immunization was most efficient when using Ccl3-fusion vaccines, and resulted in strong cytotoxic T cell responses. In contrast, Xcl1-fusion vaccines induced stronger responses after i.d. DNA immunization, which also correlated with better protection against influenza infection. In summary, our result suggest that different chemokine fusion vaccines and delivery routes may be suitable for different vaccination purposes, depending on the desired type of immune response.

## Results

### Characterization of Xcl1- and Ccl3-targeted fusion vaccines

**Figure 1:**
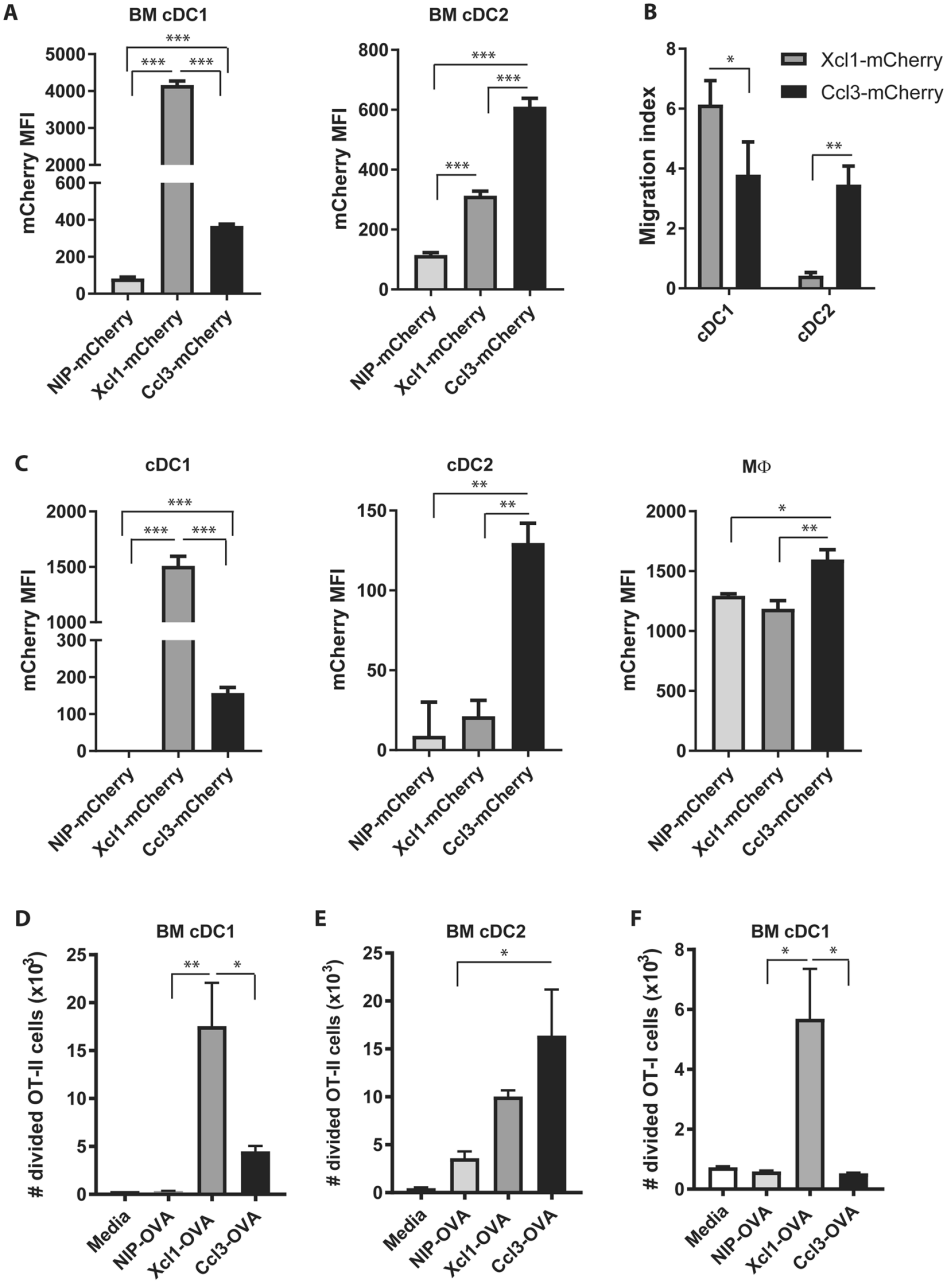
Characterization of Ccl3- and Xcl1-fusion vaccines. (**A**) BM DCs were incubated for 18 h with 0.5 μg Ccl3-, Xcl1- or anti-NIP-mCherry, and specific staining of cDC1 and cDC2 evaluated by flow cytometry after gating as indicated in Supplementary Fig. S1B. (**B**) Chemotaxis of BM DC were evaluated in transwell plates with 1.5 μg/ml Ccl3-, Xcl1- or anti-NIP-mCherry added to the bottom well. Migrated cells were identified as cDC1 or cDC2 by flow cytometry. The number of migrated cells were normalized to the number of spontaneously migrated cell when only medium was added to the bottom well. (**C**) *In vivo* targeting of APC. BALB/c mice were injected i.v. with 20 μg Ccl3-, Xcl1- or anti-NIP-mCherry. Spleens were harvested after 2 h and the mCherry staining of cDC1, cDC2 and macrophages analyzed by flow cytometry after gating as described in Supplementary Fig. S1D. (**D,E**) Proliferation of OT-II (**D**) and OT-I (**E**) cells after 4 days incubation with cDC1 or cDC2 and NIP-, Ccl3- or Xcl1-OVA. Number of proliferating cells was determined by CTV dye dilution by flow cytometry. Data shown are mean + SEM and representative of 2 independent experiments with (**A**) 6 replications or (**B,D,E**) 3 replications pr. group, or (**C**) 3 mice pr. group. Statistical analysis performed using (**A**,**C**) one-way ANOVA with Tukey’s multiple comparison test, (**B**) t-test, *p < 0.05, **p < 0.01, ***p < 0.001.

To compare immune responses against Xcl1- and Ccl3-fusion DNA vaccines, we utilized a dimeric vaccine molecule format (Vaccibody) previously constructed in our laboratory^33^. The vaccibody consists of a targeting unit (either Xcl1 or Ccl3), a dimerization unit consisting of the C_H_3 and a shortened hinge region from human IgG3 and an antigenic unit (Supplementary Fig. S1A). While fusing antigen to Xcl1 is an efficient method for selectively delivering antigen to Xcr1^+^ cDC1^6,34,35^, the surface receptors for Ccl3 are more promiscuously expressed on APC (reviewed in^31^). To evaluate how efficiently Ccl3 and Xcl1 target cDC, we generated and purified Ccl3-mCherry and Xcl1-mCherry fusion molecules and evaluated staining of bone marrow derived DCs (BM DC)^21^. Anti-NIP-mCherry (containing a scFv specific for the hapten 5-iodo-4-hydroxy-3-nitrophenacetyl (referred to as NIP)) was included as a non-targeted control^36^. While Xcl1-mCherry predominantly stained BM cDC1 (defined as CD11c^+^CD45R^−^CD24^high^), Ccl3-mCherry stained both cDC1 and cDC2 (CD11c^+^CD45R^−^CD11b^high^) to a similar degree (Fig. 1A, Supplementary Fig. S1B). There was some staining of cDC2 with Xcl1-mCherry compared to the NIP-mCherry, but this may be a results of glycosaminoglycan (GAG) binding properties of Xcl1^37^. Consistent with this interpretation, Xcl1-mCherry selectively induced chemotaxis of cDC1 in a transwell chemotaxis assay, while Ccl3-mCherry induced a similar degree of chemotaxis of both cDC1 and cDC2 (Fig. 1B). To investigate if Xcl1 or Ccl3 could directly activate cDCs, BM DCs were incubated with 0.5 μg anti-NIP-, Ccl3- or Xcl1-mCherry for 18 h and the expression of CD40, CD80 and CD86 evaluated by flow cytometry. Neither Ccl3- nor Xcl1-mCherry induced detectable activation of cDC1 or cDC2 as defined by upregulation of CD40, CD80 or CD86, which is in accordance with previous publications for Xcl1^35^ (Supplementary Fig. S1C).

To ensure targeting of cDC *in vivo* under similar conditions, Ccl3-, Xcl1- or anti-NIP-mCherry were injected i.v. into BALB/c mice and spleens harvested after 2 hours. cDCs and macrophages were gated as recently published (Supplementary Fig. S1D)^38^, and evaluated for mCherry staining. As observed *in vitro*, Xcl1-mCherry delivered i.v. specifically stained splenic cDC1, while Ccl3-mCherry stained cDC1 and cDC2 to a similar degree (Fig. 1C). For Ccl3-mCherry we also observed a slightly enhanced staining of macrophages (Fig. 1C). In summary, these results demonstrate that Xcl1-fusion vaccines selectively target cDC1, while Ccl3-fusion vaccines target both cDC1 and cDC2.

To evaluate the ability of Xcl1- and Ccl3-fusion vaccines to induce proliferation of T cells, cell trace violet (CTV) labelled OT-I and OT-II cells were incubated with sorted BM cDC1 or cDC2 in the presence of Xcl1-, Ccl3- or NIP-OVA for 4 days. Proliferation was determined by CTV dye dilution by flow cytometry (Supplementary Fig. S2A). Xcl1- and Ccl3-OVA induced significantly higher proliferation of OT-II cells compared NIP-OVA when incubated with BM cDC1 (Fig. 1D). Interestingly, Xcl1-OVA also induced higher proliferation of OT-II cells compared to Ccl3-OVA when incubated with BM cDC1. In contrast, Ccl3-OVA induce higher proliferation of OT-II cells compared to NIP-OVA when incubated with BM cDC2 (Fig. 1E). There was an increase in proliferation of OT-II seen with Xcl1-OVA and cDC2, but the difference was not significant compared to NIP-OVA. Surprisingly, only Xcl1-OVA induced proliferation of OT-I cells when incubated with cDC1 (Fig. 1F). No OT-I proliferation was seen with cDC2 incubated with either Xcl1- or Ccl3-OVA, although we did observe proliferation when the cells were incubated with the OVA derived peptide SINFEEKL as a positive control (Supplementary Fig. 2B,C).

### T cell responses after intramuscular or intradermal DNA vaccination

To compare immune responses induced by Ccl3- and Xcl1-fusion vaccines, the major surface antigen Hemagglutinin (HA) from influenza A/34/PR/8 (PR8) was used as an antigen^5,6,39^. Xcl1-HA and Ccl3-HA constructs were expressed at similar levels *in vitro* as determined by ELISA on supernatants from transiently transfected HEK293E cells (Supplementary Fig. S3A). The sizes of the expressed vaccibodies under reducing and non-reducing conditions were analyzed by SDS-PAGE, and confirmed that the vaccibodies were predominantly secreted as dimers (Supplementary Fig. S3B).

**Figure 2:**
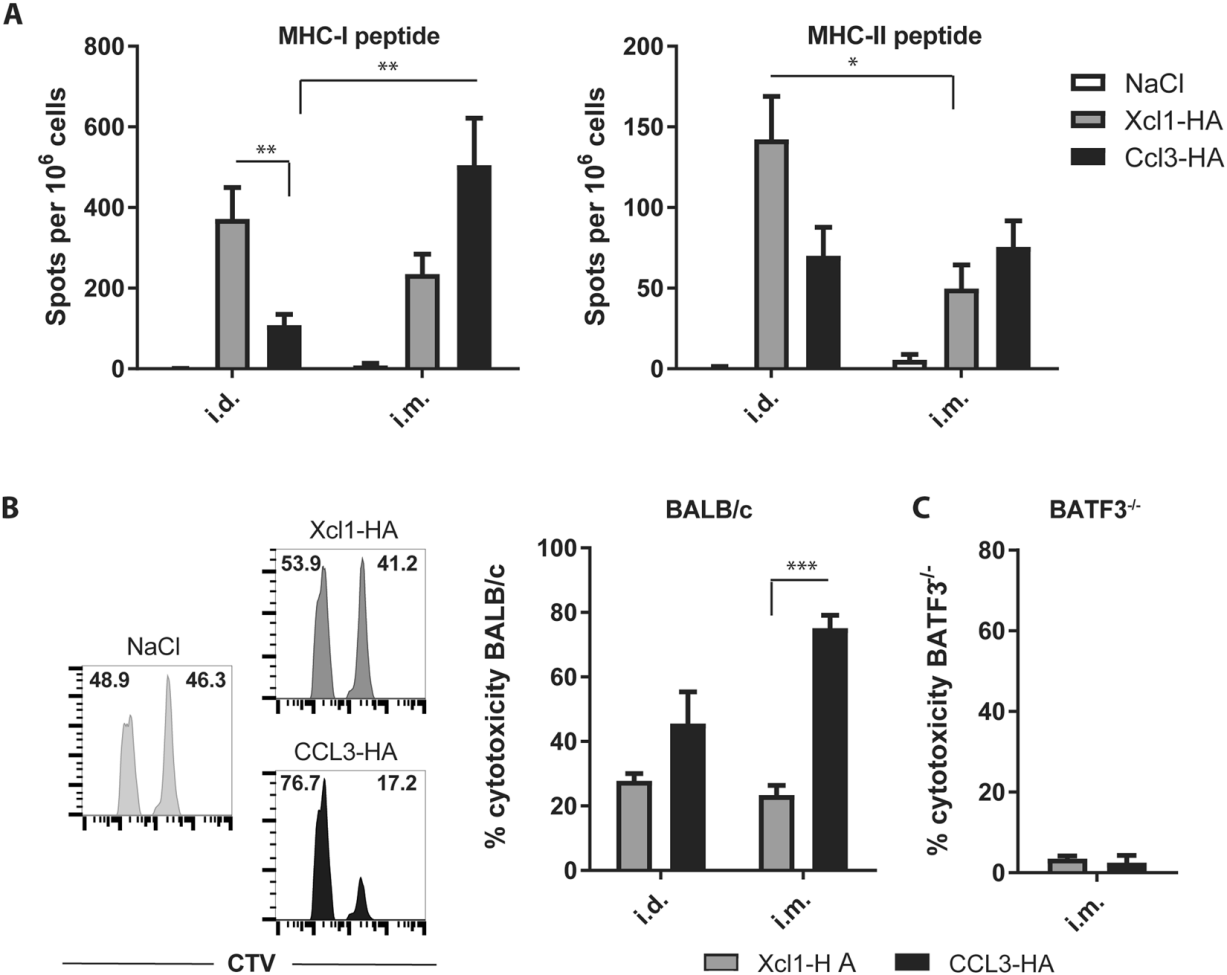
T cell responses after i.m. or i.d. DNA immunization. (**A**) IFNγ ELISPOT on splenocytes harvested from BALB/c mice 2 weeks after a single i.m. or i.d. immunization with plasmids encoding Xcl1-HA or Ccl3-HA. Splenocytes were stimulated with 2 μg/ml (left graph) IYSTVASSL (MHC-I restricted) or (right graph) HNTNGVTAACSHEG (MHC-II restricted) peptides. (**B**) *In vivo* cytotoxicity of BALB/c splenocytes pulsed with IYSTVASSL (CTV^high^) or a control peptide (DSSLQDGEFI) (CTV^low^) before i.v. injection into BALB/c mice immunized two weeks prior with Xcl1-HA or CCL3-HA by i.m. or i.d. immunization. Representative histograms after i.m. DNA immunization are dispayed on the left. Percentage of CTV^low^ and CTV^high^ cells are indicated within each histogram. The cytotoxicity data is summarized in the right graph. (**C**) Cytotoxicity assay as in (**B**) performed in BATF3 knockout mice i.m. immunized with Xcl1-HA or Ccl3-HA. (**A**) pooled from 3 independent experiments with 12–13 mice pr group, (**B**) pooled from 2 independent experiments with n = 10 mice pr group, and (**C**) data from one experiment with n = 4 mice pr group. Statistical analysis performed using non-parametric one-way ANOVA with Dunn’s multiple comparison test, *p < 0.05, **p < 0.01, ***p < 0.001.

Immune responses induced by Xcl1-HA and Ccl3-HA DNA vaccines were evaluated in BALB/c mice immunized by either i.m. or i.d. administration of plasmids encoding the fusion vaccines. To enhance uptake of DNA and subsequent immune responses, the injection site was electroporated by delivering short electric pulses using either an Elgen^40^ (i.m.) or a DermaVax^41^ (i.d.) delivery system. T cell responses were evaluated in spleens of BALB/C mice 2 weeks after a single immunization. The number of IFNγ-secreting cells were analyzed by ELISPOT after stimulation with a MHC-I restricted peptide (IYSTVASSL) or a MHC-II restricted peptide (HNTNGVTAACSHEG), as indications of CD8^+^ and CD4^+^ T cell responses, respectively. i.d. DNA immunization with Xcl1-HA induced significantly higher numbers of IFNγ-secreting CD8^+^ T cells compared to Ccl3-HA (Fig. 2A). In contrast, i.m. delivery resulted in higher number of IFNγ-secreting CD8^+^ T cells in CCL3-HA immunized mice compared to Xcl1-HA, although the difference did not reach significance. i.m. immunization with Ccl3-HA did, however, induce significantly higher numbers of IFNγ-secreting CD8^+^ T cells compared to i.d. immunization with Ccl3-HA (Fig. 2A). No significant differences were observed in the number of IFNγ-secreting CD4^+^ T cells between Xcl1-HA and Ccl3-HA immunized mice after either i.d. or i.m. delivery, although there was a tendency for Xcl1-HA to induce higher numbers after i.d. immunization (Fig. 2A). Indeed, i.d. immunization with Xcl1-HA induced significantly more of IFNγ-secreting CD4^+^ T cells compared to i.m. immunization with Xcl1-HA (Fig. 2A).

**Figure 3:**
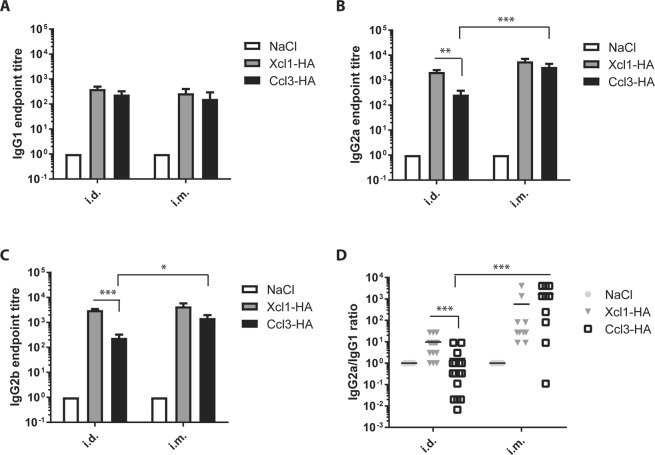
Antibody responses after a single intramuscular (i.m.) or intradermal (i.d.) DNA immunization with Xcl1- and Ccl3-fusion vaccines containing influenza virus hemagglutinin. (**A–C**) Serum titers of IgG1 (**A**), IgG2a (**B**) and IgG2b (**C**) from BALB/c mice 2 weeks after a single i.m. or i.d. immunization with plasmids encoding Xcl1-HA or Ccl3-HA. D) IgG2a/IgG1 ratio of mice presented in A and B. (**A–D**) data presented are pooled from two independent experiments with n = 12–16 mice pr group. Statistical analysis performed using non-parametric one-way ANOVA with Dunn’s multiple comparison test, *p < 0.05, **p < 0.01, ***p < 0.001.

To test for effector functions of the induced T cells, we performed an *in vivo* cytotoxicity assay. BALB/c mice were DNA vaccinated once by i.d. or i.m. immunization and injected 2 weeks later with cell trace violet (CTV) labeled splenocytes pulsed with the IYSTVASSL peptide (or a control peptide). Specific killing of the IYSTVASSL-pulsed splenocytes was analyzed after 18 hours in spleens. Surprisingly, mice immunized with Ccl3-HA displayed higher cytotoxicity compared to Xcl1-HA after both i.d. and i.m. immunization, although the difference was only significant after i.m. delivery (Fig. 3B). This observation is in contrast to the *in vitro* proliferation assay and the i.d. DNA immunization where Xcl1-fusion vaccines induced stronger CD8^+^ T cell responses compared to Ccl3-fusion vaccines. There was a tendency for Xcl1-HA to induce higher cytotoxicity after i.d. immunization, and for Ccl3-HA to induce higher cytotoxicity after i.m. immunization, but the differences did not reach significance (Fig. 3B).

cDC1s have been reported to be superior at cross-presenting antigen to CD8^+^ T cells^42^. Since Ccl3-OVA failed to induce OT-I proliferation when incubated with cDC1 *in vitro* we wanted to test if Ccl3-HA induced cytotoxic T cell responses in the absence of cDC1. We therefore repeated the cytotoxicity assay after i.m. immunization with Xcl1-HA or Ccl3-HA in BATF3^−/−^ mice that lack cDC1^43^. As expected, Xcl1-HA immunization did not induce cytotoxicity in the absence of cDC1 (Fig. 3C). More surprisingly, i.m. Ccl3-HA immunization in the BATF3^−/−^ mice also failed to induce cytotoxicity, suggesting that cDC1 are equally important for cytotoxicity when immunizing with Ccl3-fusion vaccines.

### Antibody responses after intramuscular or intradermal DNA immunization

To evaluate induction of antibodies, serum samples were harvested 2 weeks after i.m. or i.d. DNA immunization and analyzed for the presence of HA-specific antibodies of the IgG1, IgG2a and IgG2b subclasses (Fig. 3A–C). Following i.m. DNA immunization, no significant differences were observed between Ccl3-HA and Xcl1-HA for any of the three IgG subclasses, although Xcl1-HA displayed a tendency to induce higher titers of IgG2b (Fig. 3A–C). There was also no difference for the IgG2a/IgG1 ratio, with both Ccl3-HA and Xcl1-HA inducing a predominantly Th1 polarized antibody responses (Fig. 3D). As seen for the T cell responses, i.m. immunization with Ccl3-HA induced significantly higher titers of IgG2a and IgG2b compared to i.d. immunization with Ccl3-HA. Xcl1-HA however, displayed no significant differences in antibody responses after i.d. and i.m. immunization.

Similar to i.m. DNA immunization, BALB/c mice immunized by i.d. DNA vaccination displayed no difference in the induction of HA specific IgG1 between Xcl1- and Ccl3-HA (Fig. 3A). However, Xcl1-HA induced significantly higher responses of HA specific IgG2a and IgG2b after i.d. DNA immunization compared to Ccl3-HA (Fig. 2B,C), which correlates well with the higher IFNγ secreting CD4^+^ T cell responses. Consequently, mice i.d. immunized with Xcl1-HA displayed a significantly higher IgG2a/IgG1 ratio compared to Ccl3-HA, suggesting a more Th1-polarized immune response (Fig. 3D). i.m. immunization with Ccl3-HA also induced higher IgG2a/IgG1 ratio compared to i.d. delivery of the same vaccine (Fig. 3D).

### Intradermal DNA vaccination with Xcl1-HA induces superior protection against a high dose of influenza virus

**Figure 4:**
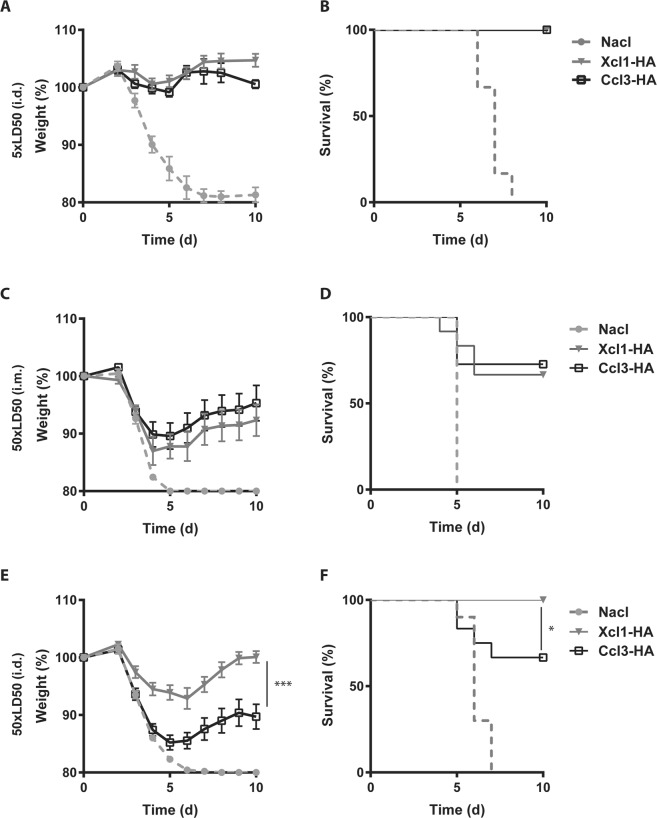
Xcl1-HA induces superior protection against a high dose of influenza virus after i.d. DNA immunization. (**A**) BALB/c mice  were challenged with 5xLD50 influenza A virus (PR8) 2 weeks after a single i.d. DNA immunization with Ccl3-HA or Xcl1-HA. Weight loss was monitored over time as a sign of disease progression. (**B**) Survival plot of the mice presented in A. (**C**) BALB/c mice were vaccinated once by i.m. DNA immunization with 50 μg plasmid encoding Xcl1-HA or Ccl3-HA and subsequently challenged with 50xLD50 influenza A virus (PR8) after 2 weeks. (**D**) Survival plot of mice presented in C. (**E**) BALB/c mice were immunized by i.d. DNA immunization with 25 μg plasmid encoding Xcl1-HA or Ccl3-HA and challenged with 50xLD50 PR8 after 2 weeks. (**F**) Survival plot of the mice presented in E. Data shown are from one single experiment with 6 mice pr group (**A** and **B**), or pooled from 2 independent experiments with 11–12 mice per group (**C–F**). Statistic analysis was performed using 2-way-anova (**A**,**C** and **E**), or Mantel Cox (**B**,**D** and **F**), *p < 0.05, ***p < 0.001.

The above results suggest that both the chemokine and the site of DNA immunization influences the immune responses. To test the protective efficacy of the targeted vaccines, we utilized an infection model where mice were challenged with a lethal dose of influenza A/PR/8/34 (H1N1) 2 weeks after immunization^6,39^. Initially, BALB/c mice were vaccinated with Ccl3*-*HA and Xcl1-HA encoding plasmids by intradermal DNA immunization and subsequently challenged with 5xLD50 PR8 virus. Mice immunized with Ccl3*-*HA or Xcl1-HA displayed only a slight weight drop after challenge and all mice survived (Fig. 4A,B). In contrast, BALB/c mice immunized with NaCl succumbed to the infection by day 8 (mice were euthanized if they lost more than 20% of their starting weight as a human endpoint).

To better differentiate the efficacy of Ccl3*-*HA or Xcl1-HA, BALB/c mice were challenged with a high dose of influenza A PR8 virus (50xLD50) two weeks after a single intramuscular or intradermal DNA immunization (Fig. 4C–F). As expected, challenging with a higher pathogen burden resulted in increased weight loss in the immunized groups, suggestive of increased morbidity. After intramuscular immunization, however, there were no difference in morbidity between BALB/c mice immunized with Xcl1-HA or Ccl3-HA (Fig. 4C), with both groups losing weight until day 5–6 and then recovering from infection. Consequently, the overall survival was similar between the two groups with 3/11 (27%) and 4/12 (33%) mice in the Ccl3-HA and Xcl1-HA groups having to be euthanized, respectively (Fig. 4D).

In contrast, BALB/c mice receiving a single i.d. vaccination with Xcl1-HA encoding plasmids displayed significantly lower morbidity compared to Ccl3-HA immunized mice when challenged with 50xLD50 PR8 virus after two weeks (Fig. 4E). Indeed, all 12 mice immunized with Xcl1-HA survived a high challenge dose, while 4/12 (33%) Ccl3-HA immunized mice had to be euthanized (Fig. 4F). Although the overall survival was similar, Ccl3-HA-immunized mice displayed significantly lower morbidity after i.m. immunization compared to i.d. immunization (Supplementary Fig. 4A,B). In contrast, i.d. DNA immunization with Xcl1-HA induced significantly lower morbidity and mortality compared to i.m. immunization with Xcl1-HA (Supplementary Fig. 4C,D).

In summary, both Ccl3- and Xcl1-fusion vaccines enhanced induction of T cell responses when given as DNA vaccines. However, the site of immunization should be taken into consideration since Ccl3 fusion vaccines generally induced more Th1 polarized antibody response with stronger cytotoxic T cell responses after i.m. immunization, while Xcl1 fusion vaccines more efficiently induced T cell responses and improved protection in an influenza mode after i.d. immunization.

## Discussion

Here we present a comparison of Ccl3-HA and Xcl1-HA DNA vaccines delivered by i.d. or i.m. injections in combination with electroporation. Our observations indicate that immunization with Xcl1-HA induced stronger cellular and antibody responses when delivered by i.d. injection, which resulted in better protection against influenza infection. In contrast, Ccl3-HA induced stronger immune responses after i.m. immunization, and induced higher cytotoxic T cell responses compared to Xcl1-HA.

There are few studies comparing i.d. and i.m. delivery of DNA vaccines in combination with electroporation. Indeed, most studies do not includes electroporation of the injection sites^23,44,45^, or only includes electroporation for one of the delivery methods^46^. Nevertheless, delivery of DNA by i.m. injection has been reported to be a more efficient method for inducing Th1 associated immune responses such as IgG2a antibodies^23,44^, in addition to inducing stronger cytotoxic T cell responses^24,44^. In contrast, delivery of DNA i.d. has been reported to induce a more Th2/IgG1 polarized response^23,44^, and more protective antibody responses^46^. Our observations with Ccl3-HA fusion vaccines correlate well with these observations, with i.m. immunization inducing stronger T cell responses and a more Th1 polarized antibody response compared to i.d. immunization with Ccl3-HA. In contrast, DNA immunization with Xcl1-HA induced a similarly Th1 polarized immune response independent of i.m. or i.d. delivery. There was a tendency for higher IgG2a/IgG1 ratio after i.m. immunization with Xcl1-HA, but the difference to i.d. immunization was not significant. Indeed, i.d. immunization with Xcl1-HA induced higher numbers of IFNγ-secreting T cells compared to i.m. immunization. Consequently, our observations indicate that Xcl1-fusion vaccines are capable of enhancing Th1 polarization independent of immunization site. Interestingly, both Xcl1 and Ccl3 have been reported to be Th1 associated chemokines that enhance Th1 polarization in combination with IFNγ^47^. However, our observations suggest that Xcl1 is more potent at promoting Th1 polarization compared to Ccl3.

Surprisingly, Ccl3-HA induced stronger cytotoxic T cell responses independent of immunization site compared to Xcl1-HA, although the difference was only significant after i.m. delivery. Considering that Xcr1 is selectively expressed on cDC1 that excel at cross-presenting antigen to CD8^+^ T cell^25,26,42^, this observations was contrary to expectations. In addition, *in vitro* proliferation of OT-I cells was also only observed with Xcl1-OVA, and not when Ccl3-OVA was incubated with cDC1. However, Ccl3-mCherry was able to target cDC2s which have been reported to efficiently cross-present antigen to CD8^+^ T cells under inflammatory conditions^48,49^. Nevertheless, the observations in BATF3^−/−^ mice, suggest that Ccl3-HA was dependent on cDC1 for induction of cytotoxicity, even when delivering DNA with electroporation that induces inflammation^50^. Ccl3 has been reported to be important for activation of CD11c^+^CD11b^+^CD8α^−^ DCs after viral infection^51^, although in our experiments, Ccl3-mCherry did not activate BM derived cDC1 or cDC2 after *in vitro* incubation. It is possible that the enhanced cytotoxicity seen with Ccl3-HA is mediated indirectly through a second cell population or additional signaling molecules. Studies by Brewitz and colleagues have indicated that pDCs can enhance CD8 T cell responses induced by cDC1, and that recruitment of CCR5^+^ pDC to CD8^+^ T cell-cDC1 complexes can be mediated via Ccl3, which could explain our observations^32^.

Although Ccl3-HA induced the strongest cellular cytotoxicity, Xcl1-HA provided better protection against a high dose challenge with influenza A virus. While it is likely that HA specific antibody responses contribute to this protection, we have previously observed that the protection mediated by i.d. immunization with Xcl1-HA is largely dependent on CD8^+^ T cells^6^. It is possible that targeting using Ccl3- and Xcl1-fusion vaccines differentially influence T cell tissue imprinting and subsequent migration. cDC1 have been reported to be important for the generation of tissue resident CD8^+^ T cell^52^, which are thought to play an important role in mediating protection against influenza hetero-subtypic infection^53^.

In conclusion, our observations suggest that DNA vaccination with plasmids encoding Xcl1- and Ccl3-fusion molecules differs in their optimal site of delivery. Since Ccl3 and Xcl1 target receptors that are differentially expressed on cDC1 and cDC2, it is possible that the frequency of different DC populations in the tissue influence the resulting immune response. Under steady state condition, however, the frequency of cDC2 has been reported to be considerably higher than cDC1 in both skin and muscle^48,54^. Electroporation has been reported to induce a massive influx of DCs, in addition to other APCs^40^, but it is unclear if this effect influences the relative frequency of cDC1 and cDC2. Of relevance, we have previously observed that delivery of Xcl1-OVA fusion protein via laserporation of the skin induce cytotoxic T cell responses and reduce tumor growth in a B16 melanoma model^34^. Together with the data presented here, these observations suggest that skin is an efficient delivery site for targeting cDC1.

Chemokine fusion vaccines are currently being tested in clinical trials, and in summary, our observations suggest that both targeting strategy and delivery site should be considered depending on the desired type of immune responses.

## Material and Methods

### Cell lines, viruses and antibodies

Human embryonic kidney (HEK) 293E cells (obtained from ATCC) were used for testing expression of HA-vaccibody proteins. Mouse anti-HA (H-36-4-52, kind gift from Siegfried Weiss) was affinity purified in the laboratory. For serum IgG ELISA, anti-mouse IgG (Fc-specific), anti-mouse IgG1-bio (clone 10.9), anti-mouse IgG2a-bio (clone 8.3) and anti-mouse IgGb-bio (clone R12-3) were used. For flow cytometric analyis, anti-CD45R (clone RA3-6B2), anti-CD11c (N418), anti-CD24 (M1/69), anti-CD11b (M1/70), anti-CD49b (DX5), anti-CD3ε (145-2C11), anti-CD19 (6D5), anti-CD64 (X54-5/7.1), anti-CD26 (H194-112), anti-F4/80 (BM8), anti-MHC-II (M5/114.15.2) and anti-CD45 (30-F11) were used. Influenza virus strains A/PR/8/34(H1N1) was kindly provided by Dr. Anna Germundsson-Hauge at The National Veterinary Institute, Oslo, Norway.

### Animals

All experimental protocols involving live vertebrates were approved and carried out in accordance with the guidelines and regulations set by the Norwegian National Animal Research Authority (NARA). Female BALB/c mice aged 6–10 weeks were purchased from Janvier, France. BATF3^−/−^ mice were purchased from The Jackson Laboratory (Stock No: 013755) and bred in-house. Mice challenged intranasally (10 µl per nostril) with influenza A/PR/8/34(H1N1) virus were euthanized if they lost more than 20% of their starting weight as a humane clinical endpoint, according to the guidelines of NARA.

### Generation and purification of targeted vaccibodies

The construction of the Xcl1-targeted and CCL3-targeted vaccibodies have been described previously^5,6^. Purification of NIP-, Xcl1- and CCL3- vaccibodies with mCherry or OVA as antigen were performed as described in Gudjonsson *et al*.^21^. In brief, HEK293E cell were transiently transfected in 5-layer BD Multi-Flasks (Corning) using Lipofectamine 2000 (Invitrogen). Supernatants were harvested after 3–4 days and applied to a column containing CaptureSelect FcXL affinity Matrix (Life Technologies) connected to an ÄKTAprime plus chromatography system (GE healthcare). Bound vaccibodies were washed with PBS and eluted using 0.1 M glycin-HCl pH 2.7. Eluted proteins were dialyzed twice against PBS, and subsequently concentrated using Vivaspin20, 50.000 MWCO cutoff columns (Sartorius).

### Generation of bone marrow derived DCs (BM DCs)

The protocol for generating BM DCs by incubation with Flt3L was originally published by Brasel and colleagues^55^, and was described in detail in our previous publication^21^. For evaluation of targeting and activation after incubation with Xcl1- or CCL3-mCherry, BM derived DCs were harvested 9 days after incubation with 0.1 μg/ml recombinant murine Flt3L and seeded at a density of 4 × 10^5^ cells/well in a 96-well tissue culture plate. BM DCs were subsequently incubated with 0.5 μg/ml of of Xcl1-, CCL3- or NIP-mCherry and harvested after 18 h and evaluated for mCherry staining or expression of CD40, CD80 or CD86 by flow cytometry.

### Chemotaxis assay

The protocol for chemotaxis assay on BM DCs was described in detail in Gudjonsson *et al*.^21^.

### *In vivo* binding of Xcl1- and CCL3-mCherry

For *in vivo* staining, 25 μg of Xcl1-, CCL3- or NIP-mCherry were injected i.v. into BALB/c mice and the spleens harvested after 2 h. Single cell suspesions were generated using a Gentlemacs dissociator (Miltenyi), and subsequently treated with Tris-Buffered Ammonium Chloride (ACT) lysis buffer and filtered through a 70 μm Nylon strainer. To efficiently differentiate cDCs and macrophages, the single cell suspensions were stained with a combination of antibodies as described by Guilliams and colleagues^38^.

### OT-I/OT-II proliferation

Bone marrow derived Flt3L DCs were identified as cDC1s and cDC2s - CD45^−^CD11c^+^CD11b^−^CD24^+^ and CD45^−^CD11c^+^CD11b^+^CD24^−^, respectively, and sorted on FACSAria IIIu (BD Biosciences, Franklin Lakes, NJ). Post-sorting purity checks showed cDC1 and cDC2 populations of >99% purity. OT-I and OT-II cells were harvested from spleens of OT-I^56^ and OT-II^57^ TCR transgenic mice, and enriched with mouse CD8^+^ or CD4^+^ T cell magnetic bead isolation kit (Miltenyi Biotec, Bergisch Gladbach, Germany), respectively. OT-I and OT-II cells were stained with 5 μM CellTrace Violet (CTV, Molecular Probes, Eugene, OR) before being incubated with sorted DCs at a DC:T cell ratio of 1:10 for OT-I cells and 1:3 for OT-II cells. All incubations were done with 1 μg/ml of protein for 4 days at 37 °C 5% CO_2_, and finally the T cells were analyzed for proliferation by flow cytometry. As a positive control, cells were incubated with 1 μg/ml OVA_257–264_ or OVA_323–339_ peptide for OT-I or OT-II, respectively.

### ELISA

ELISAs were performed as previously described^6^. In brief, ELISA plates (96-well, Costar) were coated with 2 µg/ml of inactivated PR8 influenza virus (Charles River Laboratories), and incubated ON at 4 °C. The plates were subsequently blocked with 150 µl/well blocking buffer (1% (w/v) BSA in PBS with 0.02% (w/v) NaAzide) for 1 h at RT. After washing, serum samples were diluted 1:50, and subsequently serially diluted 1:3, in ELISA buffer (0.1% (w/v) BSA in PBS with 0.02% (w/v) NaAzide). ELISA plates were incubated with serum samples ON at 4 °C. Next, plates were washed and incubated with 1 µg/ml biotinylated antibodies specific for mouse IgG1, IgG2a or IgG2b, and incubated for 1 h 37 °C. After washing, the plates were incubated with streptavidin-ALP (GE Healthcare (RPN1234V), 1:3000) for 45 min at RT, before being developed by adding 100 µl/well of substrate buffer (1 mg/ml phosphate substrate (Sigma, P4744)). After 30 min OD_405_ was measured on a Tecan Sunrise spectrophotometer. Antibody titer was defined as the highest dilution of a serum sample with OD values >(mean + 3xSD) of NaCl vaccinated mice. If OD values did not exceed that of the NaCl vaccinated mice (mean + 3xSD), the sample was given an end point titer of 1.

### Intradermal (i.d.) DNA vaccination

BALB/c mice were anesthetized with 150 ZRF cocktail (250 mg/ml of Zoletil Forte (Virbac, France), 20 mg/ml Rompun (Bayer Animal Health, GmbH) and 50 μg/ml of Fentanyl (Actavis, Germany)). After shaving the lower back, 25 µl of DNA vaccine (0.5 µg/µl in 0.9% NaCl) was injected i.d. on the left flank followed by electroporation using the DermaVax (Cyto Pulse Sciences, Inc) system with 2 pulses of 450 V/cm × 2.5 µs and 8 pulses of 110 V/cm × 8.1 ms. The procedure was repeated on the right flank.

### Intramuscular (i.m.) DNA vaccination

BALB/c mice were anesthetized as described for i.d. vaccination, before each hind leg was shaved. 50 μl of DNA vaccine (0.5 μg/μl in 0.9% NaCl) was injected i.m. into each *quadriceps femoris* muscle. Electroporation was performed immediately after injection with delivery of pulses from electrodes inserted i.m. flanking the injection site (Needle EP) by the Elgen electroporator device (Elgen, Inovio Biomedical Co.); as published by Liu and colleagues^40^, but with the electric pulses 5 × 60 ms at 50 V/400 mA and 200 ms delay.

### IFNγ ELISPOT

Single cell suspensions of splenocytes were prepared using the GentleMACS dissociator according to the manufacturers enzyme-free protocol. To detect IFNγ secreted by splenocytes, an ELISpotPLUS for Mouse IFNγ kit with pre-coated anti-IFNγ plates was used in accordance with the manufacturers protocol (MABTECH AB, Nack Straand, Sweden). In short, spleens were dissociated, treated with Tris-Buffered Ammonium Chloride (ACT) lysis buffer and filtered through a 70 μm Nylon strainer to prepare single cell suspensions. Cells were added to the plates at a concentration of 0.5 × 10^6^ and restimulated with the HA-derived peptides IYSTVASSL (MHC-I, H-2K^d^ restricted) or HNTNGVTAACSHEG (MHC-II, I-E^d^-restricted), or negative control peptide at a concentration of 2 μg/ml for 18 hours at 37 °C 5% CO_2_. Plates were automatically counted and analyzed using a CTL ELISPOT reader (CTL Europe GmbH, Bonn, Germany). Values obtained from negative control peptide wells were subtracted from values obtained from stimulation with specific peptides for each sample.

### Cytotoxicity assay

*In vivo* cytotoxicity assay were modified from Durward *et al*.^58^. In brief, splenocytes were harvested and incubated with 1 μg/ml of the influenza HA peptide IYSTVASSL or a control peptide at a density of 5 × 10^7^ cells/ml for 1 h at 4 °C before being transferred to 37 °C for an additional 30 min. Peptide-loaded cells were washed twice in PBS and labeled with either 1 μM (negative control) or 10 μM (IYSTVASSL) cell trace violet (CTV) at a density of 5 × 10^7^ cells/ml for 20 min at 37 °C. After washing twice in PBS, the cells were re-suspended in PBS at a density 5 × 10^7^ cells/ml and the two populations mixed 1:1. A total of 1 × 10^7^ mixed cells were injected i.v. into BALB/c mice immunized 14 days earlier with Xcl1-HA or CCL3-HA by either intramuscular or intradermal DNA vaccination. Spleens were harvested 18 h later, and processed into single cell suspension as described for IFNγ ELISPOT. The ratio of CTV^low^ to CTV^high^ cells were determined by flow cytometric analysis, and cytotoxicity calculated as % specific lysis = [1 − (non-transferred control ratio/experimental ratio)] × 100.

### Statistics

All statistics were calculated using Prism 6.0 software (GraphPad Software, La Jolla, CA). For all figures where multiple statistical comparisons were performed on data in the same figure, such as *in vitro* and *in vivo* binding, antibody titers, ELISPOT assays and cytotox assay, two-tailed one-way ANOVA was performed with Tukey’s or Dunn’s multiple comparison test. For chemotaxis data, a two-tailed t-test was performed. For analysis of weight curves, two-way ANOVAs were performed. For survival curves Mantel-Cox test was performed.

## Supplementary information

Supplementary information

## Data Availability

Materials, data and protocols will be made available to readers upon request to the corresponding authors.
