# Author Correction: Dendritic cell targeted Ccl3- and Xcl1-fusion DNA vaccines differ in induced immune responses and optimal delivery site

**DOI:** 10.1038/s41598-020-62688-3

**Published:** 2020-03-31

**Authors:** Anna Lysén, Ranveig Braathen, Arnar Gudjonsson, Demo Yemane Tesfaye, Bjarne Bogen, Even Fossum

**Affiliations:** 1 K.G. Jebsen Centre for Influenza Vaccine Research, Institute of Immunology, University of Oslo and Oslo University Hospital, Oslo, 0372 Norway; 2 Centre for Immune Regulation, Institute of Immunology, University of Oslo and Oslo University Hospital, Oslo, 0372 Norway

Correction to: *Scientific Reports* 10.1038/s41598-018-38080-7, published online 12 February 2019

This Article contains errors.

In the Author Contributions section,

“A.L., B.B. and E.F. designed, performed and analyzed the research. E.F. and B.B. wrote the manuscript. A.L., R.B., D.T. and A.G. performed research and analyzed the results. All authors reviewed the manuscript.”

should read:

“A.L., B.B. and E.F. designed, performed and analyzed the research. E.F., A.L. and B.B. wrote the manuscript. A.L., R.B., D.T. and A.G. performed research and analyzed the results. All authors reviewed the manuscript.”

In the Results section under subheading ‘T cell responses after intramuscular or intradermal DNA vaccination’.

“Surprisingly, mice immunized with Ccl3-HA displayed higher cytotoxicity compared to Xcl1-HA after both i.d. and i.m. immunization, although the difference was only significant after i.m. delivery (Fig. 3B).”

should read:

“Surprisingly, mice immunized with Ccl3-HA displayed higher cytotoxicity compared to Xcl1-HA after both i.d. and i.m. immunization, although the difference was only significant after i.m. delivery (Fig. 2B).”

“There was a tendency for Xcl1-HA to induce higher cytotoxicity after i.d. immunization, and for Ccl3-HA to induce higher cytotoxicity after i.m. immunization, but the differences did not reach significance (Fig. 3B).”

should read:

“There was a tendency for Xcl1-HA to induce higher cytotoxicity after i.d. immunization, and for Ccl3-HA to induce higher cytotoxicity after i.m. immunization, but the differences did not reach significance (Fig. 2B).”

“As expected, Xcl1-HA immunization did not induce cytotoxicity in the absence of cDC1 (Fig. 3C).”

should read:

“As expected, Xcl1-HA immunization did not induce cytotoxicity in the absence of cDC1 (Fig. 2C).”

Finally, in the Results section under subheading ‘Antibody responses after intramuscular or intradermal DNA immunization’.

“DNA immunization compared to Ccl3-HA (Fig. 2B,C), which correlates well with the higher IFNγ secreting CD4+ T cell responses.”

should read:

“DNA immunization compared to Ccl3-HA (Fig. 3B,C), which correlates well with the higher IFNγ secreting CD4+ T cell responses.”

