# Supplementary material for: Dendritic cell targeted Ccl3- and Xcl1-fusion DNA vaccines differ in induced immune responses and optimal delivery site
Source: Sci Rep. 2019 Feb 12;9:1820. doi: 10.1038/s41598-018-38080-7 (PMC6372594; doi:10.1038/s41598-018-38080-7)

**Supplementary Information:**

**Dendritic cell targeted CCL3- and Xcl1-fusion DNA vaccines differ in induced immune responses and optimal delivery site**

Anna Lysen, Ranveig Braathen, Arnar Gudjonsson, Demo Yemane Tesfaye, Bjarne Bogen, Even Fossum.

**Supplementary Figure Legends:**

Supplementary Figure S1: Gating strategies and characterization of CCL3- and Xcl1-fusion vaccines. (A) Illustration of vaccibody protein structure with a targeting domain consisting of CCL3 or Xcl1, a dimerization domain made up from $C_H3$ and hinge and an antigenic domain containing mCherry. (B) Gating strategy used to identify mCherry staining in BM cDC1 and cDC2 after incubation with 0.5 μg anti-NIP-, CCL3- or Xcl1-mCherry for 18h. (C) Activation of BM cDC1 and cDC2 after incubation with 0.5 mg CCL3-, Xcl1- or anti-NIP-mCherry for 18h. Expression of CD40, CD80 and CD86 was evaluated by flow cytometry after gating as indicated in (B). (D) Gating strategy used to identify splenic cDC1, cDC2 and macrophages (MΦ) by flow cytometry. Data shown are representative of 2 independent experiments with (B-C) 3 replications pr. group, or (D) 3 mice pr. group.

Supplementary figure S2: In vitro proliferation of OT-I and OT-II cells. Purified OT-II (A) and and OT-I (B) cells were incubated with sorted BM derived cDC1 or cDC2, in combination with NIP-, Xcl1- or Ccl3-OVA for 4 days. OT-II cells incubated with the peptide $OVA_{323-339}$ or OT-I cells incubated with $OVA_{257-264}$ were included as positive controls. Proliferation was determined by CTV dye dilution by flow cytometry. (C) Number of proliferating OT-I cells after incubation with cDC2s and OVA containing vaccibodies. (A-C) Data shown is representative of 2 independent experiments with n = 3 replications pr. group.

Supplementary Figure S3: Characterization of Xcl1-HA and CCL3-HA fusion vaccines. (A) Secretion of CCL3-HA and Xcl1-HA from transiently transfected HEK293E cells as determined by ELISA on supernatants harvested after 72h. (B) Western blot on supernatants from HEK293E cells transiently transfected with Xcl1-HA (X) or CCL3-HA (C) under non-reducing (-

DTT) or reducing (+DTT) conditions. As protein standard (L), a Spectra Multicolor Broad Range Protein Ladder was used. Images were captured on a Syngene G:Box, and represent the full lanes of each membrane. Blots were detected using a PR8 HA specific antibody (clone H36-4-54). Data shown are (A and B) representative of 2 independent experiments.

Supplementary Figure S4: CCL3-HA induce less morbidity after i.m. DNA immunization, while Xcl1-HA induces better protection after i.d. DNA immunization. (A-C) Comparison i.m. and i.d. immunization for the BALB/C mice presented in Figure 4 C-F. (A) Weightloss and (B) survival of BALB/C mice immunized CCL3-HA by i.m. or i.d. DNA vaccination. (C) Weightloss and (D) survival of BALB/C mice immunized with Xcl1-HA by i.m. or i.d. immunization. Data shown are pooled from 2 independent experiments with 11-12 mice per group. Statistic analysis was performed using 2-way-anova (A and C), or Mantel Cox (B and D), * = $p < 0.05$, *** = $p < 0.001$.

**Supplementary Figure S1**

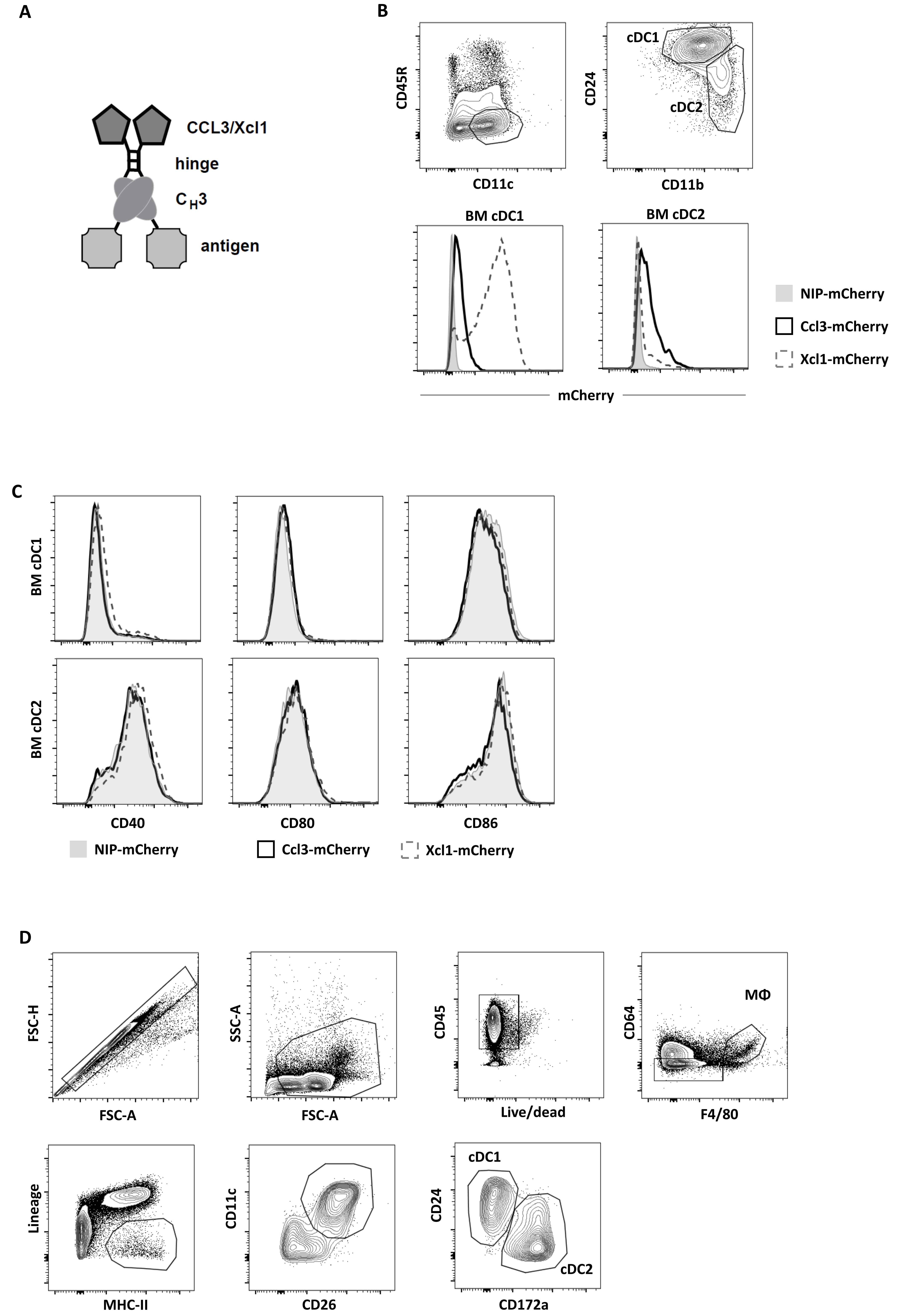

## Supplementary Figure S2

**A**

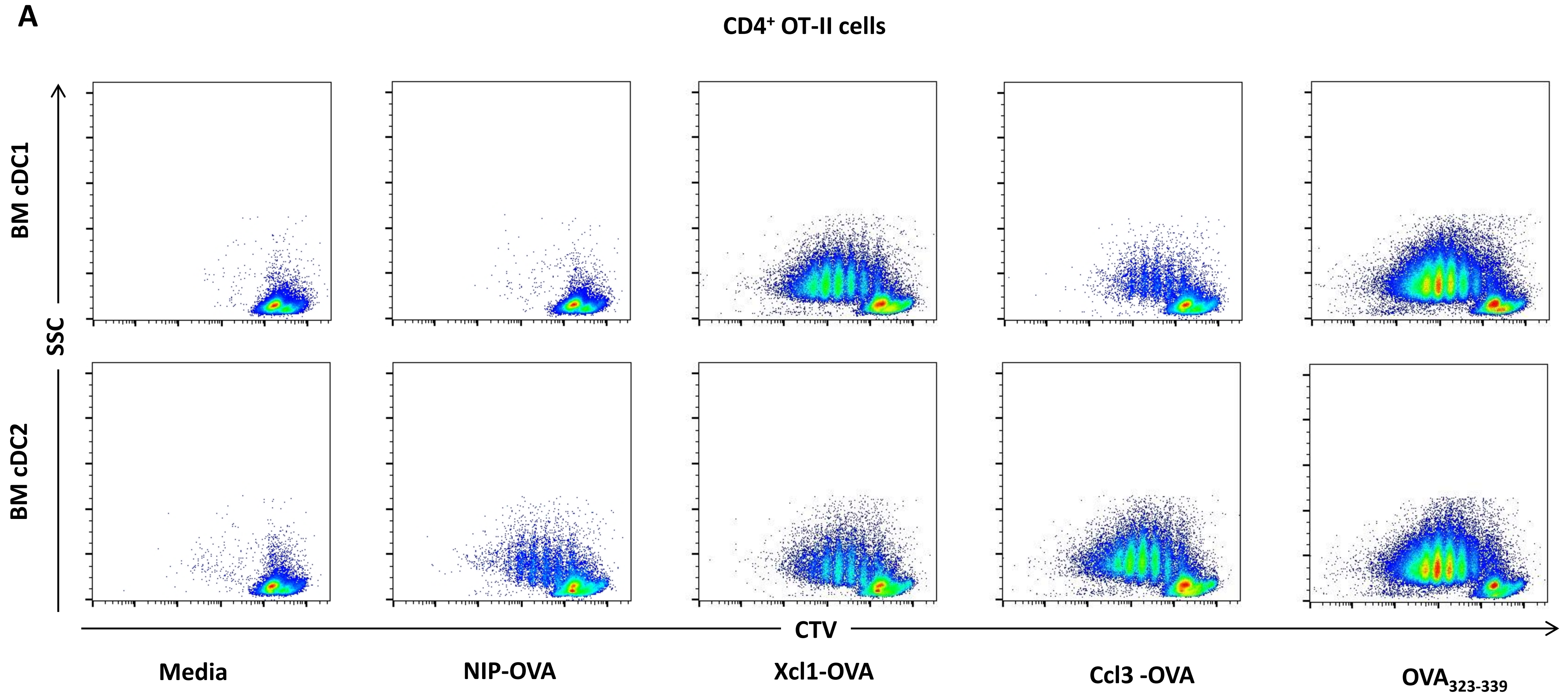


**B**

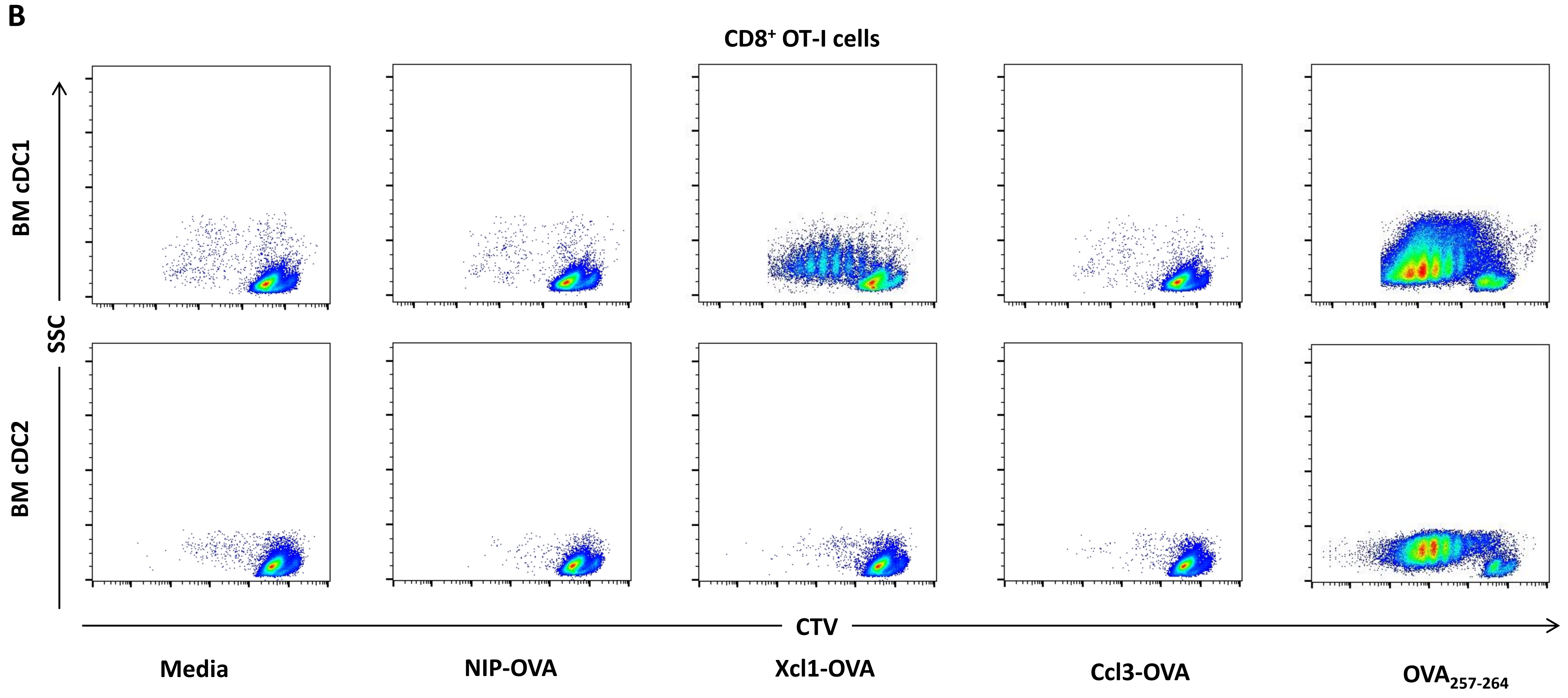


**C**

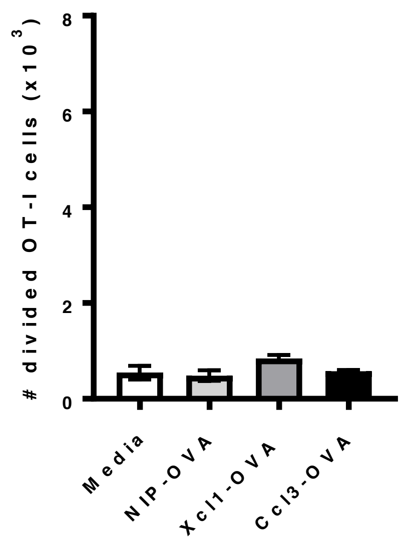

**Supplementary Figure S3**

**A**

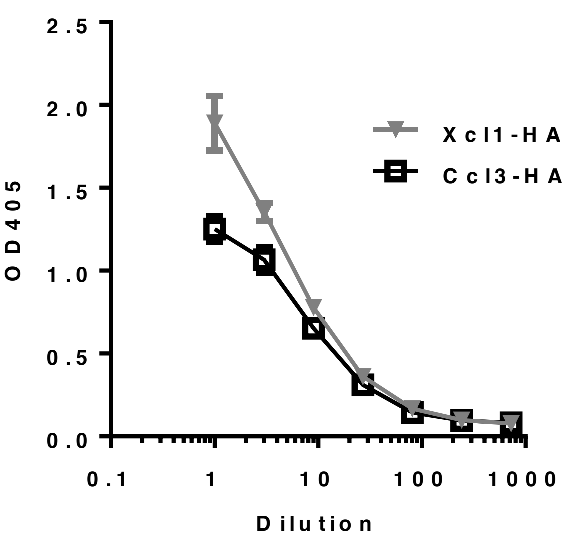


**B**

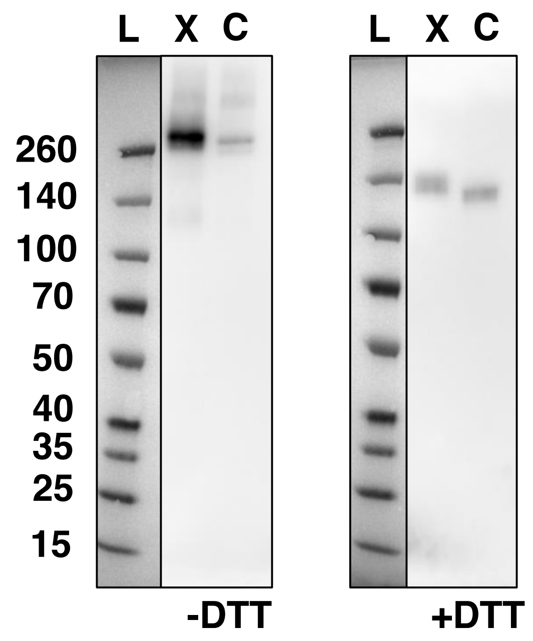

**Supplementary Figure S4**

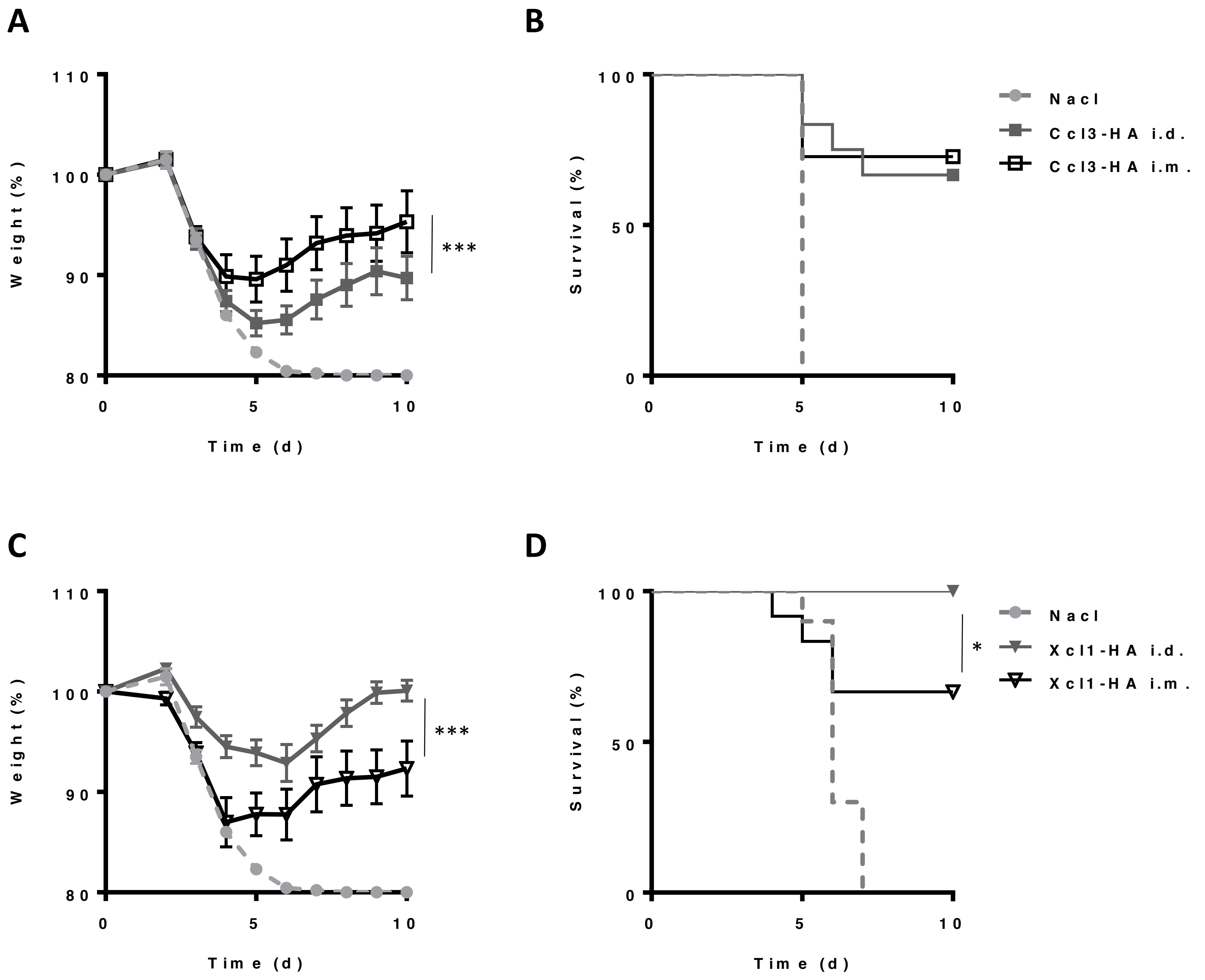

Supplement: Supplementary file 1 — Supplementary information [file 41598_2018_38080_MOESM1_ESM.pdf]
